# Unresponsive thin endometrium caused by Asherman syndrome treated with umbilical cord mesenchymal stem cells on collagen scaffolds: a pilot study

**DOI:** 10.1186/s13287-021-02499-z

**Published:** 2021-07-22

**Authors:** Yanling Zhang, Libing Shi, Xiaona Lin, Feng Zhou, Liaobing Xin, Wenzhi Xu, Huaying Yu, Jing Li, Mei Pan, Yibin Pan, Yongdong Dai, Yinli Zhang, Jia Shen, Lijuan Zhao, Min Lu, Songying Zhang

**Affiliations:** 1 Assisted Reproduction Unit, Department of Obstetrics and Gynecology, Sir Run Run Shaw Hospital, School of Medicine, Zhejiang University, NO.3 Qingchun East Road, Shangcheng District, Hangzhou, 310016 People’s Republic of China; 2 Key Laboratory of Reproductive Dysfunction Management of Zhejiang Province, Hangzhou, People’s Republic of China; 3 Department of Diagnostic Ultrasound and Echocardiography, Sir Run Run Shaw Hospital, School of Medicine, Zhejiang University, Hangzhou, People’s Republic of China; 4 Zhejiang Gene Stem Cell Biotech Co. Ltd., Hangzhou Zhejiang, People’s Republic of China

**Keywords:** Thin endometrium, Asherman syndrome, Umbilical cord mesenchymal stem cells, Collagen scaffolds, Endometrial regeneration, Cell therapy

## Abstract

**Background:**

Unresponsive thin endometrium caused by Asherman syndrome (AS) is the major cause of uterine infertility. However, current therapies are ineffective. This study is to evaluate the effect of transplantation with collagen scaffold/umbilical cord mesenchymal stem cells (CS/UC-MSCs) on this refractory disease.

**Methods:**

Eighteen infertile women with unresponsive thin endometrium, whose frozen–thawed embryo transfers (FETs) were cancelled due to reduced endometrial thickness (ET ≤ 5.5 mm), were enrolled in this before and after self-control prospective study. Hysteroscopic examination was performed to confirm no intrauterine adhesions, then twenty million UC-MSCs loaded onto a CS were transplanted into the uterine cavity in two consecutive menstrual cycles. Then uterine cavity was assessed through hysteroscopy after two transplants. FETs were performed in the following cycle. Pregnancy outcomes were followed up. Endometrial thickness, uterine receptivity and endometrial angiogenesis, proliferation and hormone response were compared before and after treatment.

**Results:**

Sixteen patients completed the study. No treatment-related serious adverse events occurred. Three months after transplantation, the average ET increased from 4.08 ± 0.26 mm to 5.87 ± 0.77 mm (*P* < 0.001). Three of 15 patients after FET got pregnant, of whom 2 gave birth successfully and 1 had a miscarriage at 25 weeks’ gestation. One of 2 patients without FET had a natural pregnancy and gave birth normally after transplantation. Immunohistochemical analysis showed increased micro-vessel density, upregulated expression of Ki67, estrogen receptor alpha, and progesterone receptor, indicating an improvement in endometrial angiogenesis, proliferation, and response to hormones.

**Conclusion:**

CS/UC-MSCs is a promising and potential approach for treating women with unresponsive thin endometrium caused by AS.

**Trial registration:**

ClinicalTrials.gov NCT03724617. Registered on 26 October 2018—prospectively registered, https://register.clinicaltrials.gov/

**Supplementary Information:**

The online version contains supplementary material available at 10.1186/s13287-021-02499-z.

## Introduction

Thin endometrium is often found in women with Asherman syndrome (AS) because the basal layer is destroyed, and the functional layer fails to respond to hormonal stimulation, which is the major cause of uterine infertility [1, 2]. Adequate endometrial thickness (ET ≥ 7 mm) at the day of embryo transplantation represents the “fertile soil” for an implanting embryo, which is essential to accomplish a successful pregnancy [3]. At present, there is no consensus on the exact definition of thin endometrium. The most widely acceptable measure is 7 mm, as an ET < 7 mm is negatively associated with the chance of implantation and pregnancy [4, 5].

Clinically, numerous strategies have been adopted to promote endometrial regeneration, including extended estrogen administration, low-dose aspirin, pentoxifylline, tocopherol, vaginal sildenafil citrate, and intrauterine perfusions with granulocyte colony-stimulating factor [6–9]. However, even with the use of these therapies, the endometrium of some patients still remained unresponsive, frozen–thawed embryo transfer (FET) cycles have to be cancelled repeatedly, or embryo implantations failed. Effective treatment for thin endometrium is still a major challenge that has not been solved, and new therapeutic approaches for increasing endometrial thickness are urgently required.

In women at reproductive age, the endometrium undergoes repeated stripping and bleeding during the menses and can be built up without scarring in subsequent cycles [10]. The regenerative capacity of the endometrium suggests that stem cells might play crucial roles in uterine homeostasis and regeneration [11]. Previous research has identified the presence of epithelial- and stromal-derived stem cells in the human endometrium [12]. Loss of endometrial stem cells might be responsible for the regeneration failure and adhesion formation in patients with AS [13]. Basic and clinical studies on the application of stem cells to treat intrauterine adhesions are well under way [14–24]. Our team demonstrated that stem cells could restore injured endometrium and improve fertility of the endometrial injury mice, which was partially attributed to angiogenesis and proliferation and macrophage immunomodulation induced by stem cells [25–27]. These results encourage us to further explore the use of stem cells for treatment of unresponsive thin endometrium caused by AS clinically. In our study, FET of all included patients repeatedly had to be cancelled due to unresponsive thin endometrium (≤ 5.5 mm). Compared with other published clinical studies [17–24], the characteristics of our study are that all patients enrolled in our study had no intrauterine adhesions and endometrial thickness was still less than 5.5 mm after hormone replacement therapies and adjuvant treatments. Besides, none of patients had history of tuberculosis (TB) infection.

Mesenchymal stromal cells (MSCs) are being investigated as a potential alternative for cellular therapy. Although bone marrow mesenchymal stem cells (BMSCs) have been used widely, the aspiration of bone marrow is invasive with relatively low cell yield, as is the use of adipose tissue stem cells (ASCs). And with increasing age, cell yield of bone marrow is significantly decreased [28]. As for MenSCs, the number available is limited because of the thin endometrium and they are easily contaminated, which might lead to endometrial inflammation, bleeding, and abdominal pain. Neonatal tissues, as a medical waste, are usually discarded without any ethical conflict and can be obtained easily, non-invasively in abundance. Another important advantage of neonatal tissues is that they provide immature cells, which have a lower risk of mutations [29]. Comparative studies of different neonatal tissues have revealed that the biological properties of MSCs from umbilical cord (UC-MSCs), chorionic plate (CP-MSCs), amniotic membrane (AM-MSCs), and placental decidua basalis (PDB-MSCs) are generally similar. However, the UC-MSCs are obtained in larger numbers and have higher proliferative potential than that of CP-MSCs, AM-MSCs, and PDB-MSCs [30, 31]. Especially the early passages of UC-MSCs are available supplies for large scale production of MSC for using in clinical studies [32]. These characteristics suggest that UC-MSCs are the best clinical tool, so we selected UC-MSCs of clinical grade for the treatment of unresponsive thin endometrium in our study.

How to transplant stem cells is an important issue to be solved in clinical applications. Tissue engineering, which involves the use of living human cells on appropriate scaffolds for the repair and reconstruction of various tissue injuries and defects, has provided a new and reliable strategy for the transplant of stem cells and has been widely recognized by the medical community [33]. Collagen scaffold with a three-dimensional structure which can guide cells to grow into the scaffold has good histocompatibility, no inflammation, and no immune rejection. In animal experiments by Guo et al., the collagen-chitosan/silica membrane dermal equivalent is beneficial to the repair of full-thickness skin burns which functions as a physical support to guide the differentiation and proliferation of cells into the targeted functional dermis [34]. Our previous studies had confirmed that collagen scaffold/umbilical cord mesenchymal stem cells (CS/UC-MSCs) could facilitate endometrial regeneration and restore fertility in rodents [25]. Here, we investigated whether transplantation of CS/UC-MSCs could expand the endometrium of patients with AS who were unresponsive to conventional treatments and thereby enhance embryo implantation and gestation.

## Materials and methods

### Isolation, identification, and differentiation of UC-MSCs

The UC-MSCs were of clinical grade, as recognized by the National Institutes for Food and Drug Control (Report number SH201702375, Supplemental Table 1) according to Chinese regulations and were provided by Zhejiang Gene Stem Cell Biotech Co. Ltd. Fresh umbilical cords (UC, *n* = 3) of normal term fetuses (maternal hepatitis B, hepatitis C virus, human immunodeficiency virus, syphilis, and other related infectious indicators are negative) were collected under sterile conditions, soaked in DMEM/F12 medium (Corning cellgro, USA, NO. 10-092-CVR), and transported on ice to the cell preparation laboratory within 48 h. UC tissues were washed with saline to remove blood stains. Residual blood, capsule, and blood vessels were removed, and the remaining tissue was cut into about 4-mm^3^ pieces. Tissue fragments were inoculated in a petri dish, cultured in DMEM/F12 medium (GIBCO, USA) supplemented with 10% fetal bovine serum (GIBCO, USA), and then placed in an incubator at 37 °C with 5% CO_2_ for 7 days. After 7 days of culture, we changed the culture medium and observed that the cells had crawled out under an inverted microscope (Olympus, Japan) and continued the culture to the 14th day. When the cell density reaches 80–90%, the primary cells were passaged and resuspended by serum-free MSC culture medium (ScienCell, Carlsbad, CA, USA, Cat. No. 7511) for use.

Flow cytometry was performed to identify the phenotype of UC-MSCs at the 3rd passage. Briefly, cells were fixed with 4% paraformaldehyde for 15 min at room temperature and blocked with 2% bovine serum albumin (BSA, Meilun Biological Technology, #MB4219, Dalian, People’s Republic of China). The cells were stained with primary antibodies, followed by fluorescein isothiocyanate-conjugated antibodies (BD, 555748) or phycoetrythrin-conjugated antibodies (BD, 555749) diluted in PBS plus 2% BSA. Stained cells (2 × 10^4^ per tube) were analyzed with BD FACSCalibur^TM^ flow cytometer (BD Bioscience, CA, USA) and analyzed using BD CellQuest^TM^ pro software version 6.0. The primary antibodies were anti-human CD105 (BD, 560839), anti-human CD73 (BD, 550257), anti-human CD34 (BD, 555822), anti-human CD45 (BD, 5554882), anti-human CD90 (BD, 555595), and anti-HLA-DR (BD,555560). For each sample, at least 10,000 events were recorded.

UC-MSCs at the 5th passage were detached by trypsin, resuspended by pre-warmed MSC culture medium, and seeded into 24-well plates at 5 × 10^4^ cells/cm^2^ for osteogenic and adipogenic differentiation and at 4 × 10^4^ cells/cm^2^ for chondrogenic differentiation. UC-MSCs were incubated at 37 °C in a humidified atmosphere of 5% CO_2_ for 24 h. MSC growth medium was replaced by osteogenic differentiation medium (Gibco, A1007201), adipogenic differentiation medium (Gibco, A1007001), and chondrogenic differentiation medium (Gibco, A1007101) in the induction groups. In the non-induction group, MSC culture medium was continued. Culture medium was changed every 3 days. The resulting calcium deposition, fat vesicles, and sulfated glycosaminoglycans were subjected to Alizarin Red (Solarbio Life Science, Beijing, China), Oil Red O (Solarbio Life Science), and Alcian Blue (Solarbio Life Science) staining 3 weeks later. For UC-MSCs extracted from one umbilical cord, all the above experiments were repeated 3 times independently.

### Ethics approval and consent to participate

This study was approved by the Ethics Committee of Stem Cell Clinical Research Institution of Sir Run Run Shaw Hospital, School of Medicine, Zhejiang University (No. 20180801-1) and conducted at the Reproductive Medicine Center of Sir Run Run Shaw Hospital from February 2019 to April 2021. All the patients signed informed consents. This study was registered on ClinicalTrials.gov (NCT03724617).

### Patients

Patients were recruited from the Reproductive Medicine Center of Sir Run Run Shaw Hospital from February 2019 to April 2020. The inclusion criteria were as follows: (1) age 20–40 years; (2) infertile patients who had received assisted reproduction treatment and had frozen embryos in store; (3) patients who underwent at least two rounds of hysteroscopic adhesiolysis (HSA) and the uterine cavity returned to normal; (4) women whose ET failed to expand beyond 5.5 mm with the use of 6–8 mg/day estradiol valerate combined with at least one round of treatment with aspirin, granulocyte colony-stimulating factor (G-CSF), heparin, vaginal sildenafil, or Chinese traditional medicine. Patients were excluded from recruitment if they had any of the following issues: (1) those who could not agree to the follow-up conditions required by the study; (2) contraindications for hysteroscopic surgery and estrogen therapy; (3) congenital uterine malformations, adenomyosis, or uterine fibroids that could impair embryo implantation; (4) chromosomal abnormalities; (5) systemic diseases such as thrombosis, cardiopulmonary diseases, hematopoietic diseases, and malignant tumors; and (6) no desire to be pregnant.

### Study design and power calculation

This study was prospective with each patient serving as her own control. We assume that the mean ET would increase from 5 to 7 mm with a standard deviation (SD) of ± 1.2 mm. Accepting type I errors (*α*) of 0.05 and type II errors (*β*) of 0.20 and assuming that the dropout rate would be 20%, the sample size should be at least 17.

### Fabrication of CS/UC-MSCs

The CS/UC-MSCs were fabricated as follows: 4 cm × 6 cm collagen scaffolds with pores of 20–200 μm in diameter (Zhenghai Biotechnology Co., Shandong, People’s Republic of China) were rinsed with serum-free MSC culture medium; excess fluid was aspirated; and a suspension of 1 × 10^7^/mL (2 mL) UC-MSCs at the 5th passage was dripped uniformly onto the scaffold. The seeded scaffolds were incubated under humid 5% CO_2_ in air at 37 °C for 1 h before transplantation.

### Hysteroscopic transplantation of CS/UC-MSCs

The CS/UC-MSC scaffold were spread onto a 10-F Foley catheter and placed into the uterine cavity. After being placed in the uterine cavity, a balloon filled with 3 mL sterile saline was inserted to assist the scaffold in attaching to the inner wall of uterine cavity. B-ultrasonography confirmed that the scaffold had adhered to the uterine wall. The patient was kept in the hospital for 2 h after this procedure to observe vital signs. The balloon was left in place for 3 days before removal. Antibiotics were used to prevent infection in all patients until 6 days after surgery.

### Study procedure

**Fig. 1:**
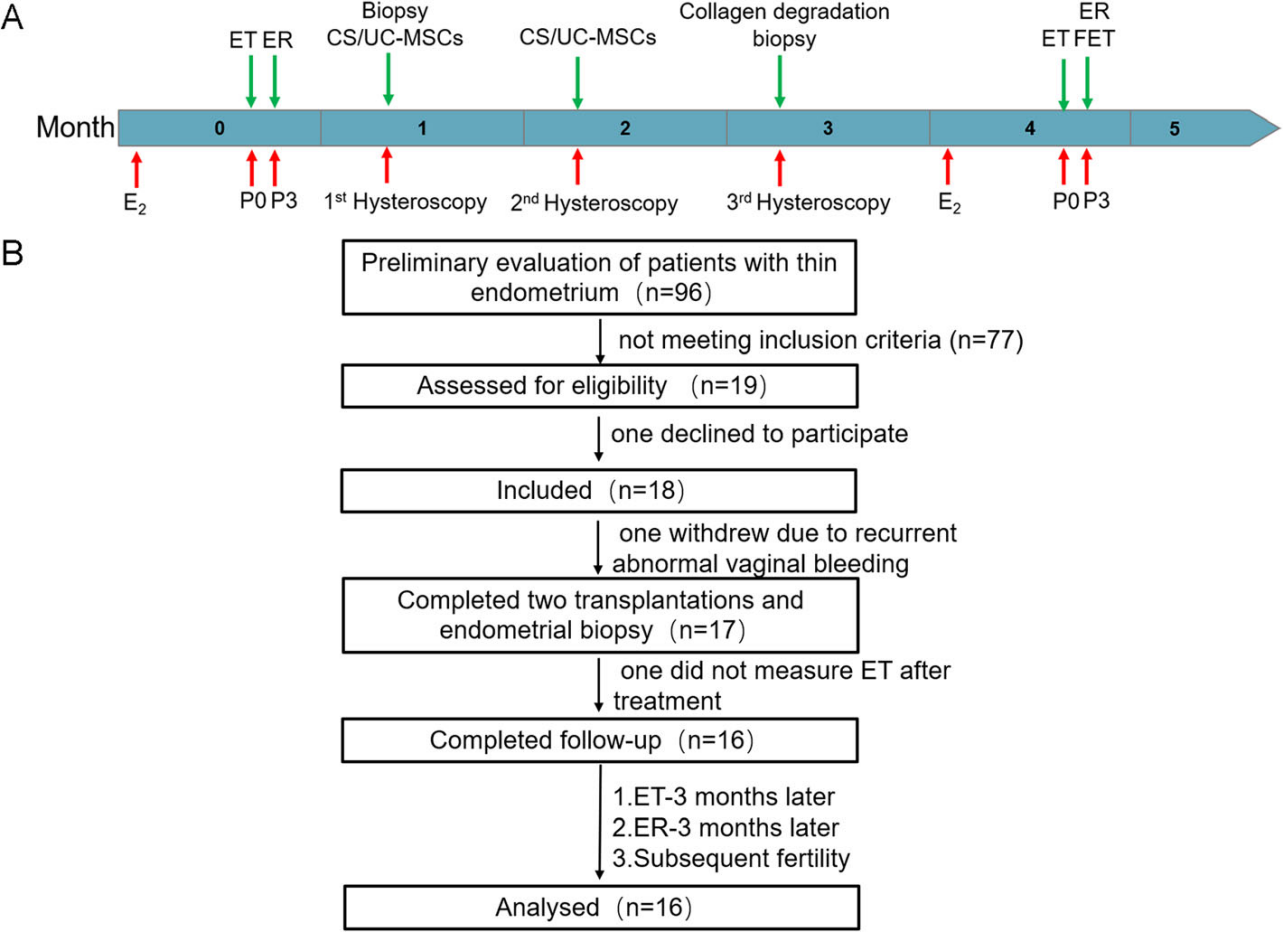
Study design and flowchart showing the patient enrollment. **A** Flowchart showing the study procedure. **B** Flowchart showing the patient enrollment. CS/UC-MSCs, collagen scaffolds/umbilical cord mesenchymal stem cells; ET, endometrial thickness; ER, endometrial receptivity; FET, frozen–thawed embryo transfer; P0, on the day of starting progesterone-based HRT

The patient underwent CS/UC-MSC transplantation on the 7–12th day of menstruation. The study procedure is outlined in the flow chart shown in Fig. 1A. Specifically, hysteroscopic CS/UC-MSC transplantation was performed twice by the same gynecologist in two consecutive menstrual cycles. We observed the uterine cavity and whether the collagen scaffold had degraded using a third hysteroscopy 1 month after these two transplant procedures. We collected endometrial biopsy specimens at the same location of the uterus at the first and third hysteroscopies. During this period, the patients did not receive any hormone therapy. After the third hysteroscopy, patients were invited to receive the hormonal replacement therapy (HRT) for FET. The HRT included estradiol valerate 6 mg/day from the third day of menstrual cycle for 12 days. A vaginal 2D ultrasound was then performed to measure endometrial thickness and to confirm a triple layer pattern. Then estradiol valerate was continued and progesterone therapy was then administered with both progesterone injection 40 mg/day, oral dydrogesterone tablets 20 mg/day, and progesterone Soft Capsules 200 mg/day for 3 or 5 days depending on the embryo’s age before embryo transfer.

### Follow-up and data collection

Patient follow-up was performed either in the clinic or by telephone consultation, ending in May 2021. Any surgical complications (e.g., uterine perforation or anesthesia accidents), and the patient’s body temperature and hemograms before and after the transplantation were recorded. The hemograms, and liver and kidney function test results were recorded 1 week after the operation. All patients were followed up to determine whether there was any tumor formation.

The primary outcome was the ET measured on the day of starting progesterone before and 3 months after surgery. Secondary outcomes included endometrial morphology under hysteroscopy, uterine receptivity, pregnancy outcomes, and endometrial histology. Ultrasonography was performed to evaluate uterine receptivity with a 5–9 MHz endovaginal probe using a GE Voluson E10 (GE Medical Systems, Milwaukee, WI, USA) by the same expert examiner at day 3 of progesterone administration during HRT cycles. The evaluation indicators for uterine receptivity in this study mainly included (1) ET; (2) endometrial volume; (3) endometrial and subendometrial blood flow, which were observed and classified using the Applebaum classification [35]; (4) uterine artery hemodynamic parameters, such as pulse index (PI), resistance index (RI), and systolic peak velocity/diastolic peak velocity ratios (S/D), which were measured as reported [36].

Pregnant women were followed up until the end of pregnancy, during which fetal conformation and aneuploidy screening and routine prenatal examinations were performed. Any placental complications were monitored by ultrasonography during pregnancy.

### Histological analysis

All endometrial biopsies were taken during the proliferative phase. Human endometrial formalin-fixed and paraffin wax-embedded biopsies obtained before and after treatment was used for hematoxylin and eosin (H&E) staining and immunohistochemistry as previously described [26]. The number of glands was recorded on H&E staining. Three fields (× 100) in each image were randomly selected for counting. The primary antibodies for immunohistochemistry used in this study included CD34, Ki67, estrogen receptor alpha (ERα), and progesterone receptor (PR) (Abcam, Cambridge, MA, USA). Images were captured and analyzed by microscopy (BX40, Olympus Optical Corporation, Tokyo, Japan). MVD was measured as described previously [26]. For the analysis of ki67, ERα and PR, a semi-quantitative grading system (H-score) was used to evaluate the intensity and percentage of staining. This was calculated as: H-score = ΣPi (*i* + 1), where *i* indicates the intensity of staining with a value of 1, 2, or 3 (weak, moderate, or strong, respectively) and Pi stands for the percentage of stained cells in the whole image, with intensity ranging from 0 to 100%.

### Statistics

Statistical analysis was performed using GraphPad PRISM software (v. 7.04; La Jolla, CA, USA). Paired *t* test was used for continuous variables. Fisher’s exact test was performed for comparing categorical variables. A two-sided *P* value of < .05 was considered statistically significant.

## Results

### Characteristic of UC-MSCs

UC-MSCs were spindle-shaped in morphology. UC-MSCs were positive for CD90, CD105, and CD73, and negative for CD34, CD45, and HLA-DR. UC-MSCs were able to differentiate into osteoblasts, adipocytes, and chondrocytes (Supplemental Figure 1 and Supplemental Figure 2).

### Participants and baseline characteristics

Between February 2019 and November 2019, after preliminary evaluation of 96 patients diagnosed with thin endometrium in our reproduction center, 77 of whom did not meet the enrollment criteria. Then 19 patients were enrolled in the study (Fig. 1B) meeting the prescribed inclusion and exclusion criteria. Seventeen patients completed the stem cell therapy, because one declined to participate, and one patient withdrew from the study because of massive recurrent abnormal vaginal bleeding (Supplemental Material). All the 17 included patients had experienced an average of 3.2 attempted hysteroscopic surgeries. Specifically, four patients experienced two hysteroscopic attempts, eight had three, two had four, and three had five. The mean age of patients was 34.1 ± 3.6 years. The mean duration of infertility was 4.2 ± 2.5 years (range 1–12 years). Thirteen patients presented with hypomenorrhea, two had amenorrhea, and two patients had normal menstrual histories (Table 1). However, among the 17 patients, 1 (patient #11) did not receive endometrial thickness measurement after treatment due to personal reasons.

**Table 1:** Clinical characteristics and outcome of patients

Patient	Age (years)	Symptoms	Etiology of AS (times)	Prior repair attempts	Score of AS at 1st HSA	Previous treatment received	ET (pre-/post-therapy mm)	Pregnancy outcome
P1	39	Infertility (3 years) Hypomenorrhea	5 D&C	3 HSA	AS Stage II	Estrogen/Aspirin/Heparin/GH/Sildenafil/traditional Chinese medicine	5.5/7.1	2 FET and implantation failure
P2	33	Infertility (3 years) Hypomenorrhea	1 D&C	3 HSA	AS Stage III	Estrogen/Aspirin/Heparin/Sildenafil	3/6.1	1 FET and implantation failure
P3	30	Infertility (4 years) Hypomenorrhea	1 HSP 1 D&C	5 HSA	AS Stage IV	Estrogen/Aspirin/Heparin/GH /Sildenafil/G-CSF/GM-CSF/traditional Chinese medicine	5.1/6.6	3 FET and implantation failure
P4	37	Infertility (3 years)	6 D&C	2 HSA	AS Stage IV	Estrogen/Aspirin/Heparin/GH /G-CSF	5.1/5.9	1 FET and implantation failure
P5	34	Infertility (3 years) Hypomenorrhea	2 D&C	2 HSA	AS Stage II	Estrogen/Aspirin/traditional Chinese medicine	5.2/5.8	1 FET 4 months post-treatment and cesarean section at 35 + 5 weeks, girl,1950 g
P6	34	Infertility (6 years) Hypomenorrhea	2 D&C	5 HSA	AS Stage IV	Estrogen/Aspirin/Heparin/GH/Sildenafil	2.3/6.1	1 FET and implantation failure
P7	35	Infertility (4 years) Hypomenorrhea	4 D&C	4 HSA	AS Stage II	Estrogen/Aspirin/Heparin/GH/G-CSF	5.1/6.4	2 FET and implantation failure
P8	35	Infertility (4 years) Hypomenorrhea	1 D&C	3 HSA	AS Stage III	Estrogen/Aspirin/Heparin	4.5/6.6	1 FET 2 months post-treatment and abortion at 25+ weeks
P9	39	Infertility (6 years) Hypomenorrhea	1 D&C	3 HSA	AS Stage IV	Estrogen/Aspirin/Heparin/GH/Sildenafil	3.7/5	2 FET and implantation failure
P10	30	Infertility (1 year) Hypomenorrhea	4 D&C	2 HSA	AS Stage III	Estrogen/Aspirin/Heparin/Sildenafil	3.5/4.5	Spontaneous pregnancy 9 months post-treatment and natural labor at 39 weeks, boy, 2600 g
P11	37	Infertility (3 years) Hypomenorrhea	3 D&C	2 HSA	AS Stage IV	Estrogen/Aspirin/Heparin	4.5/-	Did not measure ET after treatment and no FET
P12	36	Infertility (4 years) Hypomenorrhea	3 D&C	3 HSA	AS Stage III	Estrogen/Aspirin	3.4/5.8	1 FET and implantation failure
P13	31	Infertility (5 years) Hypomenorrhea	2 D&C	3 HSA	AS Stage IV	Estrogen/Aspirin/Heparin/Sildenafil/G-CSF	4.9/6.4	2 FET and implantation failure
P14	33	Infertility (5 years) Hypomenorrhea	8 D&C	4 HSA	AS Stage III	Estrogen/Aspirin/Heparin/Sildenafil	4.2/5.9	2 FET and implantation failure
P15	26	Infertility (5 years) Amenorrhea	2 D&C	5 HSA	AS Stage VA	Estrogen/Aspirin/Heparin/Sildenafil	2.5/4.7	1 FET and implantation failure
P16	37	Infertility (2 years) Hypomenorrhea	1 D&C	3 HSA	AS Stage IV	Estrogen/Aspirin/Heparin	3.5/-	Withdrew
P17	32	Infertility (1 years) Amenorrhea	1 D&C	3 HSA	AS Stage Va	Estrogen/Aspirin/Heparin	4.4/6.3	1 FET 3 months post-treatment and natural labor at 40 + 1 weeks, girl, 2900 g
P18	39	Infertility (12 years)	1 D&C	3 HSA	AS Stage IV	Estrogen/Aspirin/Heparin/G-CSF	3/4.7	1 FET and implantation failure

*P*, patient; *D&C*, dilatation and curettage; *HSP*, hysteroscopic polypectomy; *HSA*, hysteroscopic adhesiolysis; *AS*, Asherman, syndrome; *ET*, endometrial thickness; *GH*, growth hormone; *G-CSF*, granulocyte colony-stimulating factor; *FET*, frozen–thawed embryo transfer

### Adverse events and safety assessment

To assess the safety of stem cell therapy, we determined surgical complications, local and systemic safety issues after treatment. None of the patients had surgical complications such as postoperative fever after surgery. So far, no patients have developed tumors during the follow-up period. All patients had normal hemograms with average leukocyte counts of 6.69 ± 1.22 10^9^/L, lymphocyte counts of 2.37 ± 0.46 10^9^/L, neutrophils 54.76 ± 6.74%, and normal liver and kidney functions at 7 days after surgery (Supplemental Table 2). From H&E staining, no inflammation reaction was detected after transplantation (Supplemental Figure 3).

### Hysteroscopic examination

**Fig. 2:**
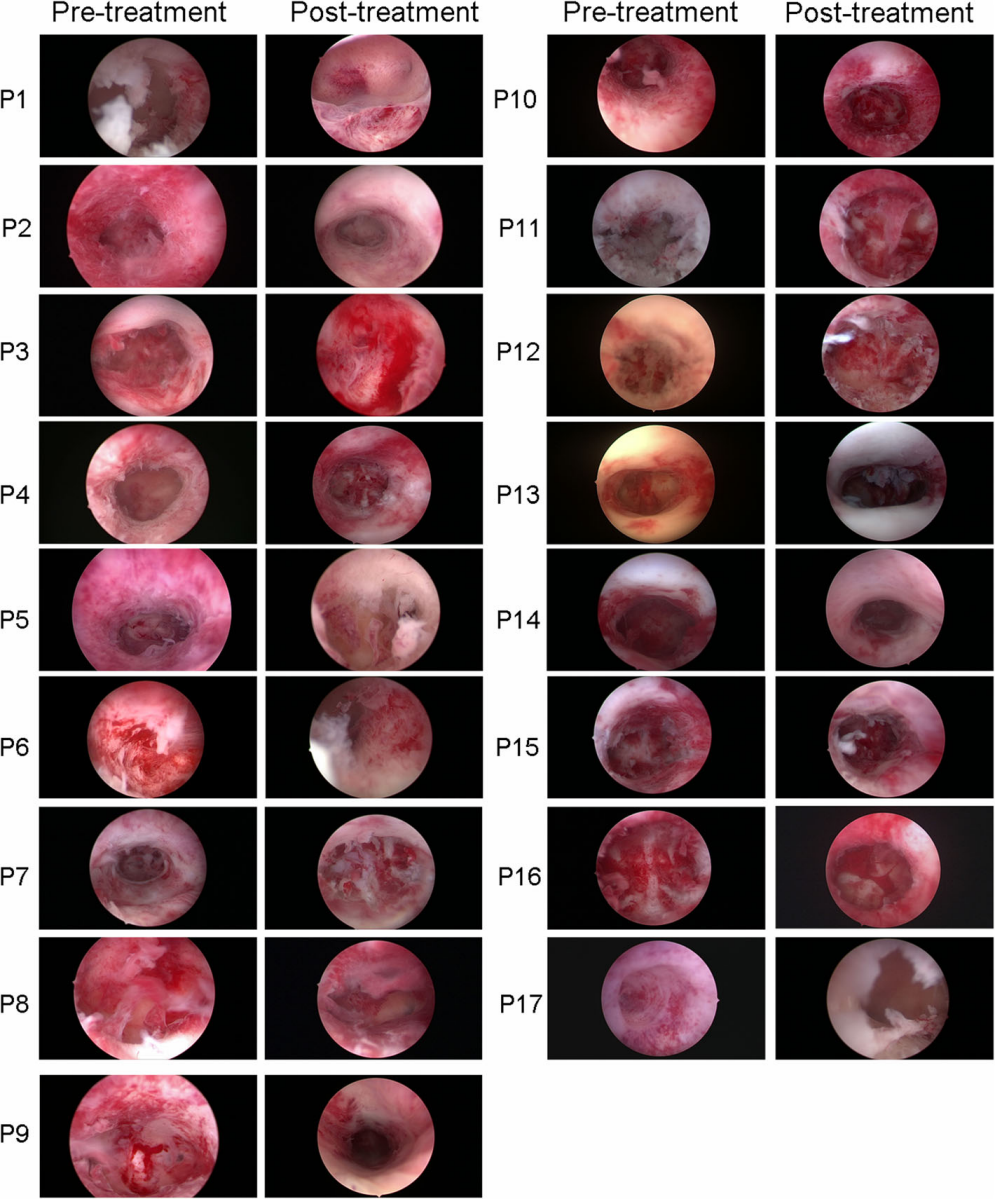
Hysteroscopy images from all 17 patients before and after CS/UC-MSC treatment

Under the first hysteroscopy, the uterine cavity of all enrolled patients appeared normal, while most of the endometrium was thin and looked rough with some scar formation. After 2 months, the endometrium demonstrated better morphology with reduced scar area under the third hysteroscopic review (Fig. 2).

### Improvement in endometrial receptivity after implantation with CS/UC-MSCs

**Fig. 3:**
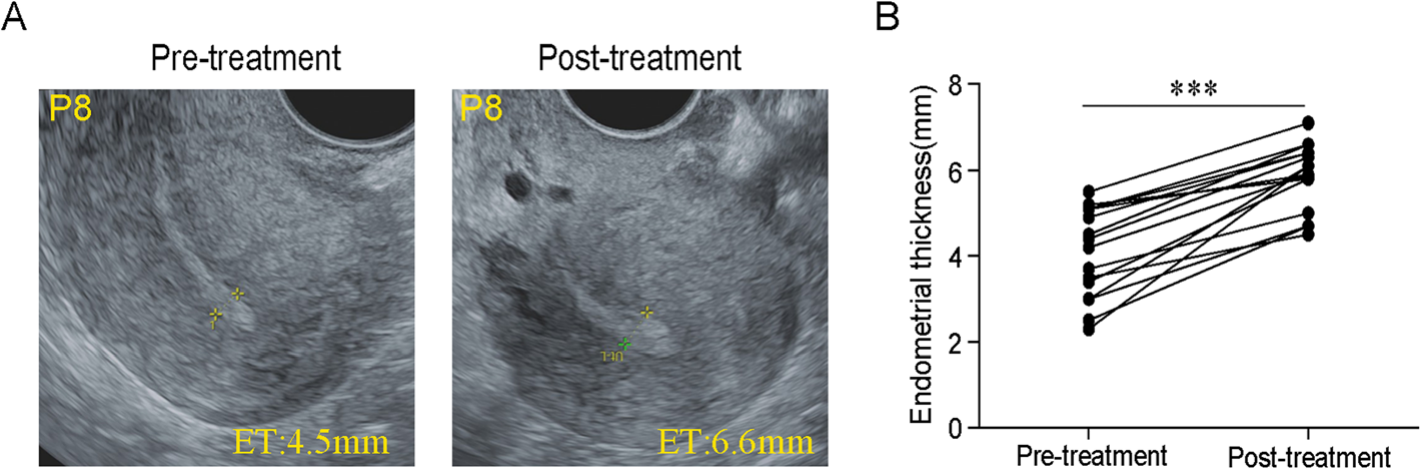
Improvement of endometrial receptivity after implantation with CS/UC-MSCs. **A** Transvaginal ultrasonography of the uterus before and after transplantation of CS/UC-MSCs in patient #8. **B** Endometrial thickness before and after transplantation of CS/UC-MSCs. Results are shown as the mean ± S.D., ***P < .001. S.D., standard deviation

Endometrial thickness has been generally considered as an important component of endometrial receptivity [37, 38]. Although our patient had no adhesions at the time of stem cell transplantation, the endometrium was scarred without a clear three-line structure under ultrasound due to severe intrauterine adhesions and could not expand to 5.5 mm after many times of HRTs and adjuvant therapies, but after adding CS/UC-MSC transplantation, the endometrial thickness was significantly increased from 4.08 ± 0.26 to 5.87 ± 0.77 mm (*n* = 16). The difference was statistically significant (*P* < 0.001, Fig. 3). The endometrial thickness of 12 patients exceeds 5.5 mm after treatment, of which 8 patients exceed 6 mm. Therefore, we believe that stem cell transplantation is beneficial to the proliferation of endometrium.

Low endometrial volumes are associated with poor pregnancy rates [39]. Kovachev et al. found that a volume of < 2 cm^3^ resulted in significantly lower implantation rates, whereas an endometrial volume of > 2 cm^3^ was a positive predictor for successful ART outcome [40]. In our study, the endometrial volume increased from 1.00 ± 0.32 to 1.12 ± 0.56 cm^3^; however, there is no significance.

The spiral artery of the uterus is the main blood vessel that nourishes the endometrium which characterized by low resistance blood flow spectrum. To a certain extent, the values of PI, RI, and S/D of the spiral artery reflect the resistance of the nourishing arterial bed. The lower the value, the higher the viability of trophoblast cells [36]. In our study, S/D measures of the uterine artery dropped from 8.03 ± 2.31 to 6.53 ± 1.21, indicating that the blood flow resistance decreased after treatment, and the ability of endometrium to accept embryos increased, while PI and RI presented no significant improvement before and after the stem cells therapy (Table 2).

**Table 2:** Evaluation of endometrial receptivity parameters

Parameters	Pre-treatment (*n* = 12)	Post-treatment (*n* = 12)	*P* value
Endometrial volume (cm^3^)	1.00 ± 0.32	1.12 ± 0.56	*P* = 0.52
Subendometrial blood flow			*P* = 0.21
Sparse	6	6	
I	6	3	
II	0	3	
Endometrial blood flow			*P* = 0.25
Sparse	9	7	
I	3	2	
II	0	3	
PI	2.66 ± 0.50	2.57 ± 0.58	*P* = 0.70
RI	0.87 ± 0.05	0.86 ± 0.06	*P* = 0.63
S/D	8.03 ± 2.31	6.53 ± 1.21	*P* = 0.06

*PI*, average value of left and right pulse index; *RI*, average value of left and right resistance index; *S/D*, average value of left and right systolic peak velocity/diastolic peak velocity ratios

### Pregnancy outcomes

By the end of December 2020, 15 of the patients had undergone 22 FETs. The patient’s embryos are listed in Supplemental Table 3. Three of these patients become pregnant, of whom two had delivered live babies with no obvious birth defects and without placental complications, and one had a spontaneous abortion at 25+ weeks. One of the two patients who did not undergo FET became pregnant naturally and had delivered a healthy boy.

### Improvements in endometrial proliferation, angiogenesis, and hormonal responses

**Fig. 4:**
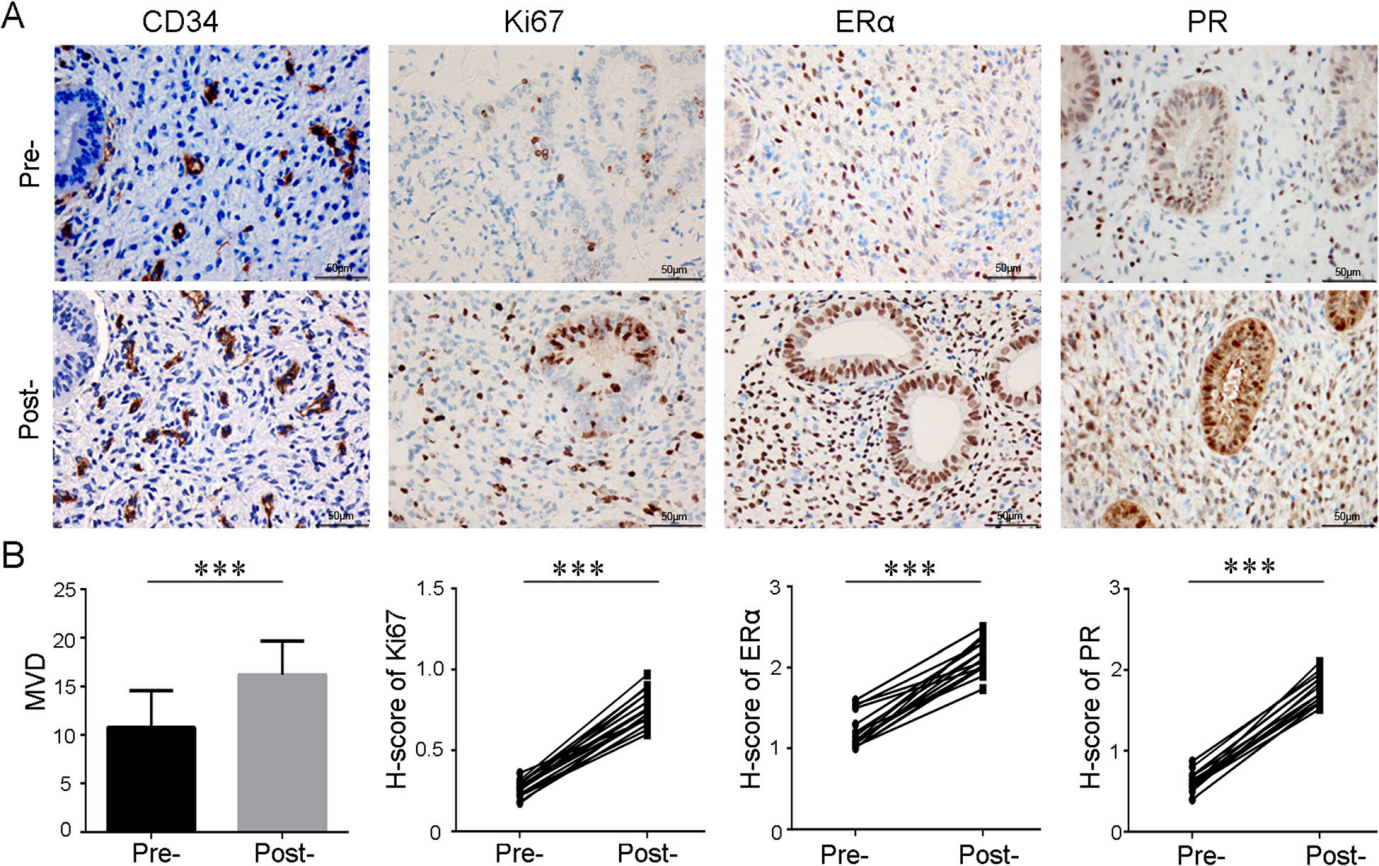
Immunohistochemical staining of CD34, Ki67, ERα, and PR on endometrial biopsy samples obtained from patients before and after CS/UC-MSC treatment. MVD was determined by CD34 immunostaining. Scale bar = 50 μm. **P < .01; ***P < .001; ERα, estrogen receptor alpha; PR, progesterone receptor; Pre-, pre-treatment; Post-, post-treatment

Stem cells can promote the proliferation of endometrial epithelial and stromal cells, thereby upregulating the expression of ERα and PR, which in turn further promote endometrial cell proliferation and vascular reconstruction [41]. Therefore, we compared MVD, and the expression of Ki67, ERα, and PR of endometrium in 17 patients before and after treatment. These molecular markers were all increased (Fig. 4), indicating that CS/UC-MSC transplantation promoted proliferation and angiogenesis in the endometrium, and enhanced its biological response to hormones. In addition, from H&E-stained images, the number of glands increased significantly after stem cell therapy (7.6 ± 0.7 to 9.2 ± 0.7/per field, Supplemental Figure 3).

## Discussion

**Fig. 5:**
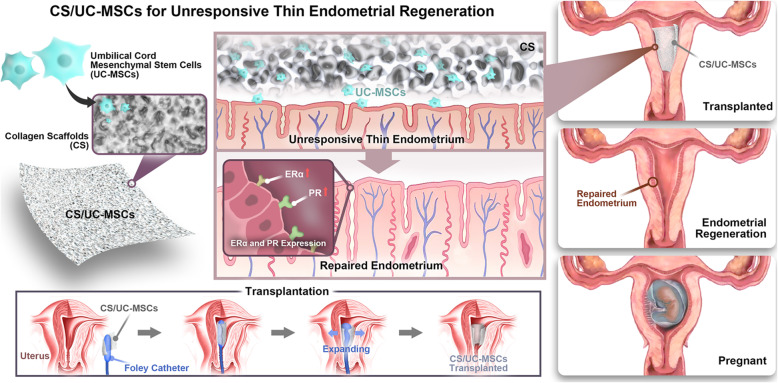
Schematic illustration of applying CS/UC-MSCs for endometrial regeneration and fertility restoration

In summary, our study is the first time to explore the effect of CS/UC-MSCs in unresponsive thin endometrium patients whose uterine cavity had returned to normal after hysteroscopic adhesiolysis (Fig. 5). Additionally, we performed two rounds of CS/UC-MSC transplantation for all patients, which is also not reported. In our study, 17 infertile patients with unresponsive thin endometrium caused by AS received CS/UC-MSC transplantation and were followed up for 2 years. There was a significant increase in endometrial thickness after the therapy. In addition, transplantation with CS/UC-MSCs could increase MVD and the expression of Ki67, ERα, and PR in endometrium, indicating that CS/UC-MSCs contribute to endometrial angiogenesis, proliferation, and differentiation.

No treatment-related serious adverse events occurred. Patient #16 withdrew from the study because of massive recurrent abnormal uterine bleeding after the first CS/UC-MSC transplantation (Supplemental Material). After multidisciplinary discussions of experts’ committee, it was concluded that “there was no direct evidence that this patient’s abnormal uterine bleeding was related to the stem cell therapy and the possibility of anovulatory abnormal uterine bleeding cannot be ruled out.” based on the following aspects: (1) We performed hysteroscopic examination to rule out surgical vessel injury. (2) The patient’s infection, bleeding, and coagulation markers were normal in the whole process. (3) The bleeding could be controlled by contraction medicine and intrauterine tamponade, stopped by progestin. However, it was reported the MSC transplantation improved angiogenesis, which may increase the tumor growth and metastasis [42]. We could not rule out the role of angiogenesis on this abnormal uterine bleeding case due to the small number of cases. A considerable number of studies still need to evaluate the safety of CS/UC-MSC therapy.

Our main outcome measure was ET. The ET from 16 patients increased from 4.08 ± 0.26 to 5.87 ± 0.77 mm on average. Endometrial thickness of patient #6 showed the most increase of 3.8 mm, from 2.3 to 6.1 mm. Although this patient was not pregnant, this increase of the endometrium was meaningful considering that our patients all had refractory thin endometrium and did not respond to conventional treatments. Patient #1 is the only patient whose ET is greater than 7 mm, but she still has implantation failure. Endometrial thickness is an important factor affecting embryo implantation, but it is not the only factor. Other influencing factors include embryo quality, age, body mass index, smoking, stress, and so on [43]. Maternal age has a significant impact on embryo quality and the success of subsequent implantation [44]. Some studies believe that with the increase of maternal age, the incidence of aneuploidy increases, while the pregnancy rate decreases [45]. Another study showed that the implantation rate of blastocyst transplantation was significantly lower and the biochemical pregnancy rate was significantly higher in women over 35 years old compared with that under the age of 35 [46]. Patient #1 was 39 years old and received twice embryo transfers after treatment, with two general embryos each time. We inferred that the implantation failure may be more related to the patient’s age and embryonic development potential.

The secondary outcomes included uterine receptivity, changes in endometrial biological indicators, and pregnancy outcomes. Endometrial volume, endometrial-subendometrial vascularization, and uterine artery blood flow did not improve significantly after therapy. The major reason is probably due to the insufficient sample size. In this study, only 12 patients received ultrasound examination on the 3rd day of progesterone-based HRT use. On the other hand, ultrasonography itself has limitations in evaluating endometrial function and is only suitable for guiding embryo transfer in a very favorable uterus or for cancelling it in extremely poor cases [47]. In addition, endometrial MVD and the expressions of Ki67, ERα, and PR after treatment were upregulated, indicating that stem cells could promote proliferation and angiogenesis in the endometrium, and enhance the biological response of the endometrium to hormones, consistent with our previous studies [25, 26]. The number of glands can also indicate the regeneration and proliferation of the endometrium. In our study, we found that the number of endometrial glands increased significantly after stem cell treatment. This result is consistent with related reports [48, 49].

Functional repair of the endometrium is mainly reflected in pregnancy outcome. In this study, 4 of the 17 patients became pregnant, of which 3 women gave birth successfully, and 1 had a miscarriage. Considering the different inclusion criteria from other studies, the pregnancy rate and the live birth rate are not comparable. In our study, all enrolled patients had no intrauterine adhesions after hysteroscopic adhesiolysis, but FET of these patients repeatedly had to be cancelled due to reduced endometrium (≤ 5.5 mm) after hormone supplements combined with adjuvant treatments. Given that, the pregnancy rate (24%) and live birth rate (18%) are acceptable.

In 2011, Nagori et al. reported the first case of AS treated with adult autologous stem cells via intrauterine infusion for endometrial regeneration that resulted in conception after in vitro fertilization and embryo transfer (IVF–ET) [17]. Eight clinical studies from six research centers have explored the effect of stem cells in treating intrauterine adhesions and endometrial atrophy [17–24], of which one was case report and seven were prospective studies (Table 3). What distinguishes our study from other studies is the inclusion of patients. In our study, all enrolled patients have no intrauterine adhesions at the time of stem cell transplantation, but ET was still less than 5.5 mm after conventional treatment and adjuvant therapy. In four of the seven published prospective studies, the intrauterine adhesions still existed at the enrollment. Hysteroscopic adhesiolysis was required and stem cells were transplanted immediately after the uterine adhesions were separated. Due to the lack of a control group for simple hysteroscopic adhesiolysis, so it is impossible to distinguish the respective roles of surgery and stem cell therapy [19, 21–23]. Therefore, our results better reflected the therapeutic effect of stem cells, and avoided the false positives possibly caused by uterine surgery. Moreover, genital TB was the most common etiology of treated patients in two studies by Singh et al. [18, 23]; all three pregnancies were in the group that underwent dilatation and curettage and none in the TB groups. Therefore, whether the inadequate growth of endometrium seen in these two studies was because of a history of endometrial TB cannot be confirmed.

**Table 3:** Summary of research on human stem cell therapy for thin endometrium

Author	Year	Number of patients	Type of stem cell	Intervention	Method of stem cell administration	ET (pre-/post-therapy)	Pregnancy outcome
Nagori et al. ^a^	2011	1 AS	Auto-BMSCs	Stem cell therapy	Intrauterine infusion	3.2/7.1	8 weeks
N Singh et al. ^b^	2014	6 AS (5/6 genital TB)	BM-MNCs	Stem cell therapy	Subendometrial zone injection	1.38/4.05/5.46/5.48	N/A
X Santamaria et al. ^b^	2016	11 AS 5 EA	Auto-CD133 + BMSCs	HSA + stem cell therapy	Uterine spiral arterioles	IUA improve obviously. EM for AS:4.3/6.7 EM for EA:4.2/5.7	2 babies born, 2 ongoing pregnancy, 2 miscarriage, 1 ectopic, 3 biochemical pregnancy
Jichun Tan et al. ^b^	2016	7 AS	Auto-MenSCs	Stem cell therapy	Intrauterine infusion	EM :3/7	2 babies born, 1 ongoing pregnancy
Guangfeng Zhao et al. ^b^	2017	5 AS	Auto-MNCs	HSA + stem cell therapy	Loaded onto a collagen scaffold	IUA improve obviously. EM:4.5/7.2	5 babies born
Yun Cao et al. ^b^	2018	26 AS	UC-MSCs	HSA + stem cell therapy	Loaded onto a collagen scaffold	IUA score:9.12/5.52 EM:4.46/5.74	8 babies born, 1 ongoing pregnancy, 1 miscarriage
Se Yun Lee et al. ^b^	2019	6 AS	Auto-ADSCs	Stem cell therapy	Intrauterine infusion	EM:3.0/6.9	1 miscarriage
N Singh et al. ^b^	2020	12 AS (9/12 genital TB) 13 EA (6/13 genital TB)	BM-MNCs	HSA + stem cell therapy	Subendometrial zone injection	IUA improve. EM for AS:2.6/4.2/4.6 EM for EA:3.6/5.9/6.5	3 babies born (all have no genital TB)

^a^Case report. ^b^Prospective study. *AS*, Asherman syndrome; *EA*, endometrial atrophy; *BMSCs*, bone marrow mesenchymal stem cells; *MNCs*, mononuclear cells; *MenSCs*, menstrual endometrial stem cells; *ADSC*, adipose-derived stem cells; *IUA*, intrauterine adhesions

Another important difference was in the frequency of stem cells administered. A single stem cell transplantation was performed in almost all patients in the seven studies. Interestingly, in Tan’s study, if the patient’s endometrium exceeds 7 mm after the first transplantation, the second one would be performed, which indicated that increasing the frequency of stem cell transplantation is beneficial to endometrial regeneration [20]. Taking into account that the endometrial thickness in our study was refractory and unresponsive, the treatment we adopted is to perform two stem cell transplantations for all patients during two consecutive menstrual cycles. Since our study is in its experimental phase, we did not compare the difference between a single transplantation and two transplantations in order to ensure the therapeutic effect of patients. Before stem cells are actually used in clinics and standard operating procedures are formed, future research needs to involve dosing frequency to standardize stem cell-based therapy.

Last but not least, the way stem cells were administered is different. In the above trials, four transplantation methods including intrauterine perfusion, spiral arterioles injection, basal layer injection, and stem cells loaded on collagen scaffold were used as shown in Table 3. The shortcomings of intrauterine perfusion are that the stem cell suspension is easy to lose, with low retention and survival rates, so the long-term treatment effect is not ideal [22]. Stem cells injected by spiral arterioles were less homed to the endometrium, thus the utilization rate of stem cells was reduced and the therapeutic effect was weakened [22]. The same situation was also observed when the SCs were injected via the tail vein in a mouse model [26]. Using the subendometrial zone injection method, it is difficult to locate the stem cells into the correct endometrial layer [22]. Compared with the other three modes of administration, UC-MSC loading on collagen scaffolds reduces the loss of stem cells and maintains a high density of stem cells in the endometrial layer to promote endometrial regeneration, which is conducive to the establishment of standardized stem cell treatment procedures. In addition, our published data showed that collagen scaffold itself does not have a better repair effect than the natural repair in promoting endometrial regeneration of rat, but the CS/UC-MSCs group is significantly better than the CS group and the natural repair group [25], indicating the increase of ET after CS/UC-MSC transplantation in this study is attributed to UC-MSCs instead of collagen.

The mechanism involved in the restoration of functional endometrium by stem cells is mainly differentiation, angiogenesis, immunosuppression, and anti-inflammatory via paracrine function [14, 50]. We have demonstrated that CS/UC-MSCs could promote proliferation and inhibit apoptosis of human endometrial stromal cell in vitro and in vivo by secreting a variety of soluble factors which have important roles in endometrial regeneration [25]. Another study from our center also confirmed that MenSCs can promote endometrial angiogenesis and regeneration through paracrine effect [26]. In the present study, endometrial MVD, proliferation index Ki67, ERα, and PR all increased significantly, indicating that the possible mechanism of such therapy is to increase endometrial angiogenesis, proliferation, and differentiation mainly by secreting abundant cytokines within the regenerative environment instead of differentiation, which is supported by Cao et al [22].

According to the above, this study has some limitations such as lack of control, a small sample size, and knowledge of underlying mechanism. Further studies should be carried out to verify the treatment effect of CS/UC-MSCs in unresponsive thin endometrium in a larger sample or RCT study and to clarify the underlying mechanism.

## Conclusions

In summary, our work describes that transplantation of CS/UC-MSCs is a promising and potential approach for treating women with unresponsive thin endometrium caused by AS. UC/MSCs may be beneficial for endometrial proliferation and angiogenesis and enhancing the response of endometrium to hormones.

## Supplementary Information

**Additional file 1: Supplemental Figure 1**. Phenotypic analysis and differentiation experiments were performed to characterize the UC-MSCs. A. Morphology of UC-MSCs at the 5th and 15th passage. B. Flow cytometry analysis showed that the positive rates of CD90, CD105 and CD73 were 99%, 99.3%, and 99%, respectively, while the hematopoietic markers CD34, CD45 and HLA were negative, identifying the cells as mesenchymal stem cells with good homogeneity. C. After 21 days of induction culture, Capacity to differentiate into osteocytes, adipocytes and chondrocytes was evaluated staining with Alizarin Red, Oil-red O and Alcian blue.

**Additional file 2: Supplemental Figure 2**. Osteogenic differentiation, adipogenic differentiation and chondrogenic differentiation of UC-MSCs in the non-induction and induction groups.

**Additional file 3: Supplemental Figure 3**. H&E pictures of 17 patients after before and after treatment.

**Additional file 4: Supplemental Table 1**. Biological safety and biological activity analysis of the clinical-grade cells^**$**^ recognized by the National Institutes for Food and Drug Control (NIFDC).

**Additional file 5: Supplemental Table 2**. Leukocyte counts, neutrophil percentage and lymphocyte count, liver function and kidney function 7 days after operation in each patient.

**Additional file 6: Supplemental Table 3**. Summary of transferred embryos of 15 patients.

**Additional file 7. Supplemental Material of the patient #16**.

## Data Availability

The datasets used and/or analyzed during the current study are available from the corresponding author on reasonable request.

## Abbreviations

CS: Collagen scaffold
UC-MSCs: Umbilical cord mesenchymal stem cells
CP-MSCs: Chorionic plate-mesenchymal stem cells
AM-MSCs: Amniotic membrane-mesenchymal stem cells
PDB-MSCs: Placental decidua basalis-mesenchymal stem cells
ET: Endometrial thickness
AS: Asherman syndrome
FET: Frozen–thawed embryo transfer
HRT: Hormone replacement therapy
HSA: Hysteroscopic adhesiolysis
G-CSF: Granulocyte colony-stimulating factor
SD: Standard deviation
ER: Endometrial receptivity
PI: Pulse index
RI: Resistance index
S/D: Systolic peak velocity/diastolic peak velocity ratios
ERα: Estrogen receptor alpha
PR: Progesterone receptor
MVD: Microvascular density
PBS: Phosphate-buffered saline
IVF–ET: In vitro fertilization and embryo transfer
D&C: Dilatation and curettage
TB: Tuberculosis
BMSCs: Bone marrow mesenchymal stem cells
ASCs: Adipose tissue stem cells
MenSCs: Menstrual endometrial stem cells
