# Supplementary figures and images for: Unresponsive thin endometrium caused by Asherman syndrome treated with umbilical cord mesenchymal stem cells on collagen scaffolds: a pilot study
Source: Stem Cell Res Ther. 2021 Jul 22;12:420. doi: 10.1186/s13287-021-02499-z (PMC8296628; doi:10.1186/s13287-021-02499-z)

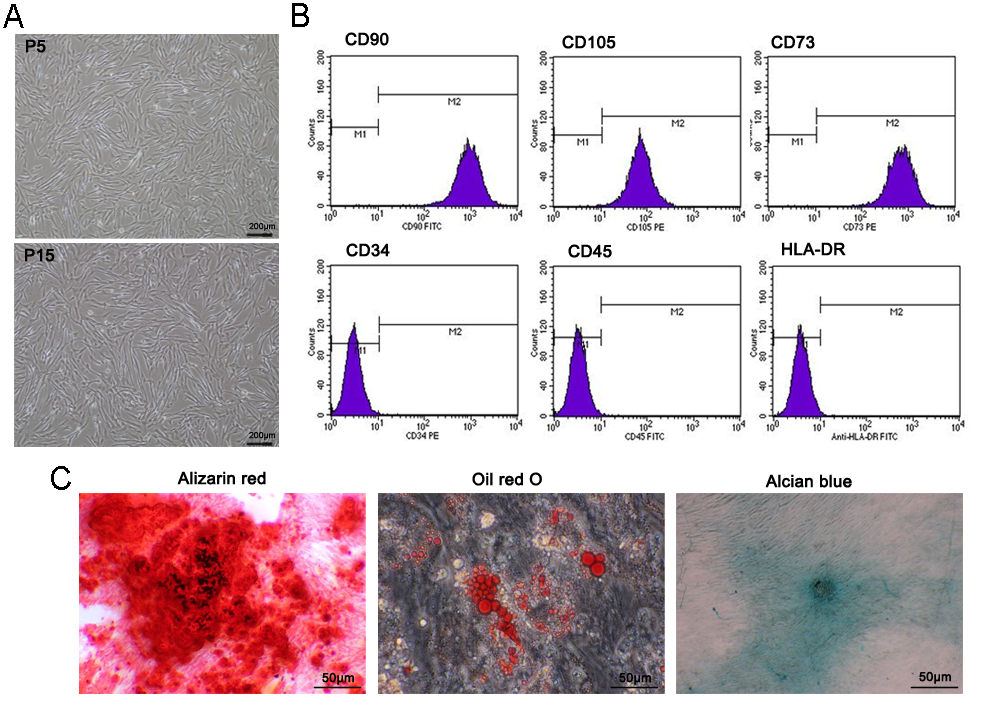

Supplement: Supplementary file 1 — Additional file 1: Supplemental Figure 1. Phenotypic analysis and differentiation experiments were performed to characterize the UC-MSCs. A. Morphology of UC-MSCs at the 5th and 15th passage. B. Flow cytometry analysis showed that the positive rates of CD90, CD105 and CD73 were 99%, 99.3%, and 99%, respectively, while the hematopoietic markers CD34, CD45 and HLA were negative, identifying the cells as mesenchymal stem cells with good homogeneity. C. After 21 days of induction culture, Capacity to differentiate into osteocytes, adipocytes and chondrocytes was evaluated staining with Alizarin Red, Oil-red O and Alcian blue. [file 13287_2021_2499_MOESM1_ESM.tif]

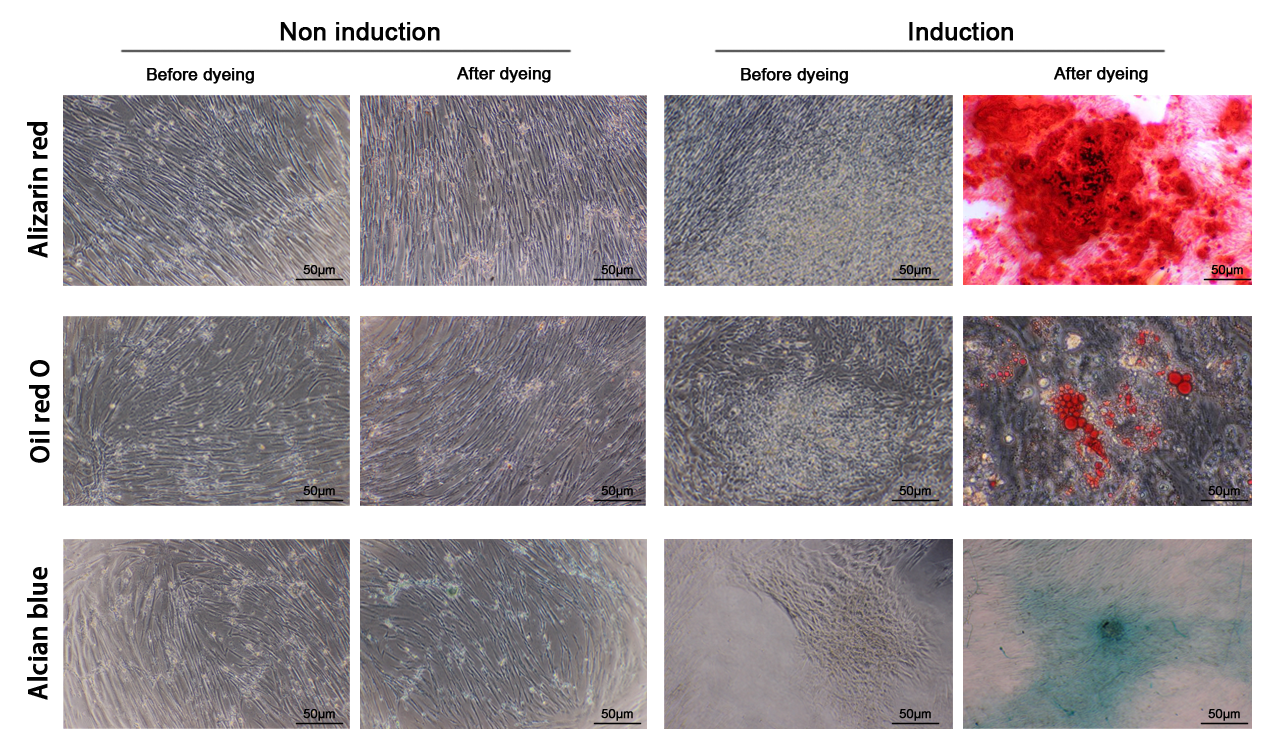

Supplement: Supplementary file 2 — Additional file 2: Supplemental Figure 2. Osteogenic differentiation, adipogenic differentiation and chondrogenic differentiation of UC-MSCs in the non-induction and induction groups. [file 13287_2021_2499_MOESM2_ESM.tif]

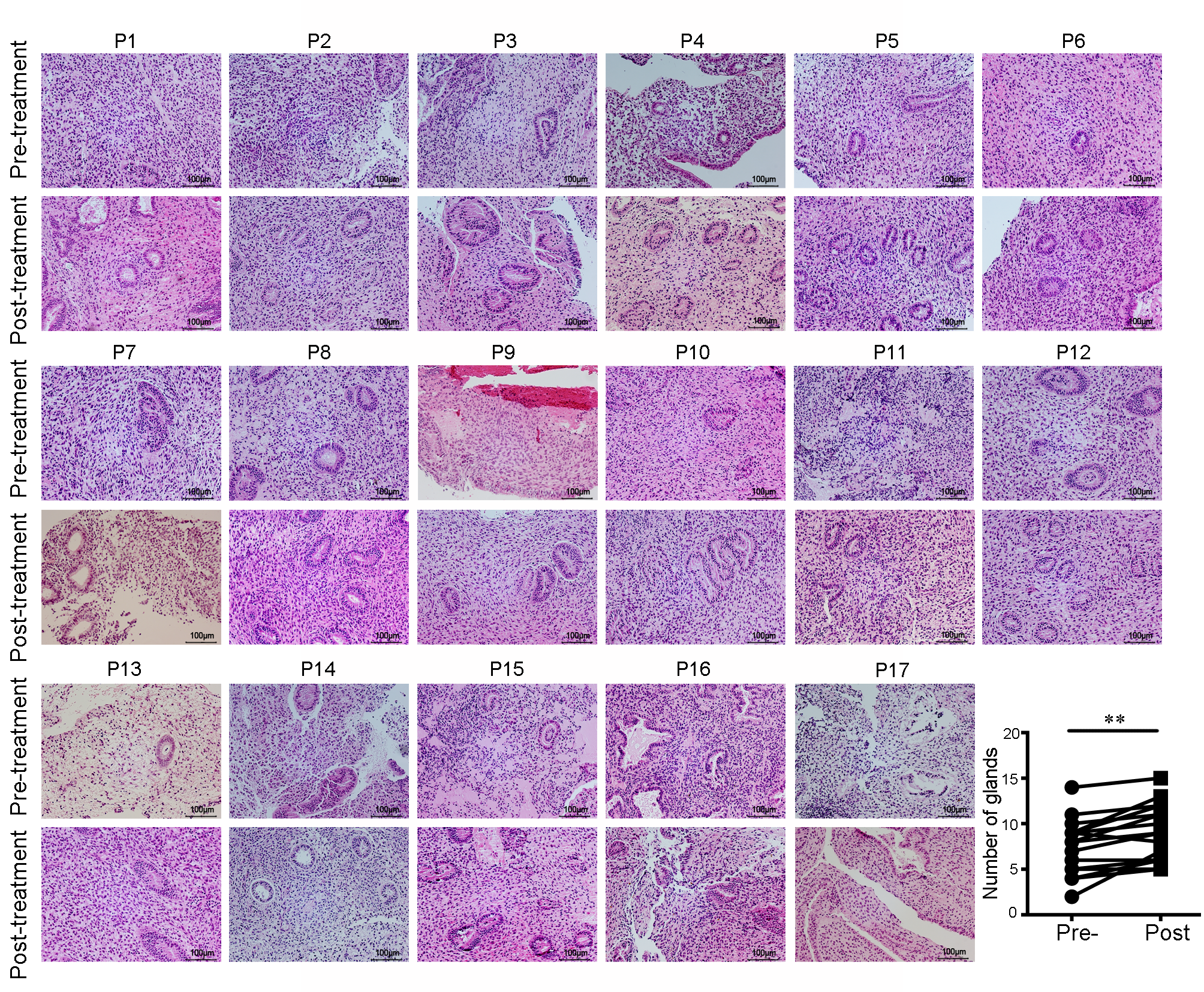

Supplement: Supplementary file 3 — Additional file 3: Supplemental Figure 3. H&E pictures of 17 patients after before and after treatment. [file 13287_2021_2499_MOESM3_ESM.tif]
