# Supplementary material for: Unresponsive thin endometrium caused by Asherman syndrome treated with umbilical cord mesenchymal stem cells on collagen scaffolds: a pilot study
Source: Stem Cell Res Ther. 2021 Jul 22;12:420. doi: 10.1186/s13287-021-02499-z (PMC8296628; doi:10.1186/s13287-021-02499-z)
Supplement: Supplementary file 4 — Additional file 4: Supplemental Table 1. Biological safety and biological activity analysis of the clinical-grade cells$ recognized by the National Institutes for Food and Drug Control (NIFDC). [file 13287_2021_2499_MOESM4_ESM.docx]

Supplemental Table S1. Biological safety and biological activity analysis of the clinical-grade cells**^$^** recognized by the National Institutes for Food and Drug Control (NIFDC)

|  | Contents |  |
| --- | --- | --- |
| Cell Characteristics | Cell morphology | Adherent growth, fibroblast morphology |
|  | Short tandem repeats (STRs) | Each STR locus has 1-2 alleles |
|  | Cell viability | 97.7% |
|  | Cytokine expression | PGE2 8.5ng/ml  HGF 2.6ng/ml  IL-6 976.8pg/ml |
| Tumorigenicity test | Detection of telomerase activity (TPG/10000 cells) | 2.4 |
| Biological activity analysis | \| [Cell surface antigen analysis](http://www.baidu.com/link?url=_pzeZ-1a76OgdTnwavSfJ1FaypDxu2YndCV-i7odQHohYxm75IfMErZh09sEEjKnzoYtN65mJxRFo8GLQy6_Ru6yAPJVzB5lGiy3H1EH6Lyxi85wgTfyPtxDwSA7y8R-yUgIW7ePNfppA89Q5QAuza) \| \| --- \| | CD73 99.4% CD90 99.8% CD105 98.5%  CD11b、CD19、CD34、CD 45、HLA-DR≤2% |
|  | Cell cycle analysis | Sub-G1 0.9% G0/G1 92.9% S 2.4% G2/M 4.7% |
|  | immunophenotypic properties | Lymphocyte proliferation inhibition ration 89%  Th1 proliferation inhibition ration 35.3%  Th17 proliferation inhibition ration 11.7%  Treg proliferation promotion ration 53.4%  Lymphocytes secreted TNF-αwith an inhibitory ration of 91.8% |
|  | Cell differentiation ability | Differentiated into adipocytes, osteoblasts in vitro |
| Biological safety analysis | Bacteria and fungi | - |
|  | Mycobacterium | - |
|  | Mycoplasma | - |
|  | Human immuno deficiency virus I (HIV- I) | - |
|  | Human hepatitis B virus (HBV) | - |
|  | Human hepatitis C virus (HCV) | - |
|  | Human cytomegalovirus (HCMV) | - |
|  | Epstein-barr virus (EBV) | - |
|  | Human papilloma virus (HPV) | - |
|  | Human herpes virus-6 (HHV-6) | - |
|  | Human herpes virus-7 (HHV-7) | - |
|  | Bovine virus | - |
|  | Porcine virus | - |
|  | Reverse transcriptase activity | - |
|  | Species identification and cell cross-contamination between species | - |
|  | Intracerebral and intraperitoneal injections in suckling mice | Survival rate 100% |
|  | Intracerebral and intraperitoneal injections in mice | Survival rate 100% |
|  | Survival rate of 5- to 6-day-old chick embryos | Survival rate 100% |
|  | Survival rate of 9- to 11-day-old chick embryos | Survival rate 100% |
|  | Intraperitoneal injection in guinea pigs | Survival rate 100%, no anatomical changes were observed at the end of the period |
|  | Hemagglutination test of 9- to 11-day-old chick embryo allantoic fluid | - |

***$*** This table is translated from NIFDC report numbers, SH201702376 for UC-MSC
