# Supplementary material for: Unresponsive thin endometrium caused by Asherman syndrome treated with umbilical cord mesenchymal stem cells on collagen scaffolds: a pilot study
Source: Stem Cell Res Ther. 2021 Jul 22;12:420. doi: 10.1186/s13287-021-02499-z (PMC8296628; doi:10.1186/s13287-021-02499-z)
Supplement: Supplementary file 5 — Additional file 5: Supplemental Table 2. Leukocyte counts, neutrophil percentage and lymphocyte count, liver function and kidney function 7 days after operation in each patient. [file 13287_2021_2499_MOESM5_ESM.docx]

| Patients NO. | leukocyte count(10^9/L) | Neutrophil percentage | lymphocyte count (10^9/L) | ALT  (U/L) | AST  (U/L) | Cre  μmol/L | BUN  mmol/L |
| --- | --- | --- | --- | --- | --- | --- | --- |
| 1 | 3.9 | 45.4 | 1.7 | 10 | 16 | 51 | 4.3 |
| 2 | 6.4 | 57.6 | 2.1 | 32 | 25 | 61 | 3.5 |
| 3 | 5.6 | 47.5 | 2.5 | 9 | 13 | 53 | 6.5 |
| 4 | 6 | 52.4 | 2.2 | 11 | 14 | 42 | 3.9 |
| 5 | 6.9 | 50.1 | 3 | 25 | 24 | 57 | 4.3 |
| 6 | 8.2 | 63.3 | 2.7 | 11 | 18 | 68 | 5.6 |
| 7 | 6.6 | 52.4 | 2.4 | 23 | 23 | 58 | 3.3 |
| 8 | 6.7 | 60.1 | 1.6 | 18 | 13 | 45 | 3.7 |
| 9 | 5.9 | 58.7 | 1.9 | 16 | 20 | 59 | 3.8 |
| 10 | 6 | 40 | 2.9 | 20 | 24 | 59 | 4 |
| 11 | 6.3 | 65.3 | 1.7 | 10 | 17 | 66 | 3.6 |
| 12 | 6.4 | 60.1 | 2.1 | 9 | 15 | 58 | 3.6 |
| 13 | 6.3 | 58.1 | 2.2 | 12 | 17 | 58 | 4.2 |
| 14 | 7.9 | 58 | 2.7 | 15 | 18 | 49 | 3.1 |
| 15 | 8.8 | 61.3 | 2.7 | 32 | 16 | 55 | 3.7 |
| 16 | 5.9 | 47.6 | 2.4 | 11 | 18 | 44 | 2.4 |
| 17 | 8.2 | 54 | 2.9 | 29 | 26 | 59 | 3.8 |
| 18 | 8.4 | 53.8 | 3 | 15 | 16 | 43 | 4 |
| ALT, alanine aminotransferase; AST, aspartate aminotransferase; Cre, Creatinine; BUN, blood urea nitrogen | | | | | | | |

Supplemental Table 2. Leukocyte counts, neutrophil percentage and lymphocyte count, liver function and kidney function 7 days after operation in each patient
