# Supplementary material for: Unresponsive thin endometrium caused by Asherman syndrome treated with umbilical cord mesenchymal stem cells on collagen scaffolds: a pilot study
Source: Stem Cell Res Ther. 2021 Jul 22;12:420. doi: 10.1186/s13287-021-02499-z (PMC8296628; doi:10.1186/s13287-021-02499-z)
Supplement: Supplementary file 6 — Additional file 6: Supplemental Table 3. Summary of transferred embryos of 15 patients. [file 13287_2021_2499_MOESM6_ESM.docx]

| Patients | Transfer times | Embryos transferred |
| --- | --- | --- |
| P1 | 2 | 1st:7-10%-2.5、8-15%-2.5  2nd:8-25%-2.5、6-10%-2.5 |
| P2 | 1 | 7-8%-2、8-8%-2 |
| P3 | 3 | 1st: 7-0%-2、8-5%-2  2nd: 7-0%-2、10-5%-2  3rd:10-8%-2/4BB (Day6) * |
| P4 | 1 | 4CB (Day5) |
| P5 | 1 | 6-15%-2.5/3CB * |
| P6 | 1 | 8-3%-2, 8-6%-2, 9-3%-2 |
| P7 | 2 | 1st:8-15%-2.5, 8-10%-2  2nd:7-20%-2.5, 6-25%-2.5 |
| P8 | 1 | 8-8%-2, 8-18%-2.5 |
| P9 | 2 | 1st:8-10%-2  2nd:4CB,4CB (Day6) |
| P12 | 1 | 8-2,8-2 |
| P13 | 2 | 1^st^:8-5%-2  2^nd^:4BB (Day5) |
| P14 | 2 | 1^st^:5BB (Day5)  2^nd^:5BB(Day5) |
| P15 | 1 | 4BB (0pn) |
| P17 | 1 | 8-8%-2 |
| P18 | 1 | 8-1, 6-3%-2 |
| P, Patient. *, 2-step embryo transfer | | |

SUPPLEMENTAL TABLE 3. Summary of transferred embryos of 15 patients
