# Supplementary material for: Unresponsive thin endometrium caused by Asherman syndrome treated with umbilical cord mesenchymal stem cells on collagen scaffolds: a pilot study
Source: Stem Cell Res Ther. 2021 Jul 22;12:420. doi: 10.1186/s13287-021-02499-z (PMC8296628; doi:10.1186/s13287-021-02499-z)
Supplement: Supplementary file 7 — Additional file 7. Supplemental Material of the patient #16. [file 13287_2021_2499_MOESM7_ESM.docx]

**Detailed treatment information of abnormal uterine bleeding patient**

The patient had a menstrual cycle of 25-26 days and was performed cesarean section in 2008. Before CS/UC-MSCs transplantation, the preoperative routine examinations are all normal, such as blood cell count, coagulation function, D-dimer, thyroid function, prolactin, antiphospholipid antibody etc.

Hysteroscopic stem cell transplantation was performed on September 24, 2019 (last menstruation: September 14, 2019). During the operation, the shape of the uterine cavity was normal, the endometrium was thin, and a scar defect was visible on the anterior wall, without uterine adhesions. After a mild scratch of the endometrium for endometrial biopsy, CS/UC-MSCs scaffold was inserted into the uterine cavity, pressed by 10 F Foley catheter filled with 3ml water. The operative process went smoothly with 1mL bleeding. There was no obvious discomfort after the operation, no fever, no abdominal pain. A little vaginal bleeding lasted for 5 days, and the oral antibiotic was given to prevent infection until 6 days after surgery. One week after the operation (October 1, 2019), the Foley catheter was taken out. The process went smoothly without bleeding. The blood routine and coagulation function were normal after a week after the operation, and the pathological results showed the proliferative phase endometrium.

The patient's first menstruation after hysteroscopic CS/UC-MSCs transplantation was October 12, 2019. The patient has less bleeding on the first day of menstruation. At 9:00 am on October 13, 2019, the patient felt heavy menstrual flow, accompanied by blood clots, no fever or night sweats, no nausea, vomiting or diarrhea, no abdominal pain, no frequent urination or dysuria, no hematuria or other discomforts. The result showed that the uterus is filled with suspension fluid, mainly in the lower part of the uterine cavity. The patient received “a single dose of oxytocin (10iu) and Agkistrodon hemagglutinin (2U) injection, and misoprostol (400ug) sublingual administration every 3 hours for three times” to promote contractions and hemostasis in the local hospital. After treatment, the amount of bleeding decreased significantly.

The large amount vaginal bleeding reoccurred next morning. The hemoglobin dropped to 72g/L, platelet count was 198×10^9^/L, prothrombin time was 12.9 seconds, and activated partial coagulation time (APTT) was 22.2 seconds. Supportive treatments such as promotion of contractions, hemostasis, and infusion were given.A balloon was placed in the uterine cavity for tamponade. The bleeding was decreased again after these management.

At 6 a.m. on October 15, 2019, hemoglobin of this patient was 63g/L and 4iu of suspended red blood cells were transfused to correct anemia. At 2 p.m. on October 15, 2019, recheck hemoglobin of this patient was 94g/L. The uterine balloon was taken out on the same day, and there was no obvious vaginal bleeding.

On the morning of October 17, 2019, a large amount of blood clots was discharged again, the hysteroscopic examination was performed to rule out surgical vessel injury. Blood clots were seen in the uterine cavity, diffuse oozing blood was seen after removal of the clots, no obvious pulsatile bleeding and no residual collagen scaffold was seen. On the same day, recheck Hb 89g/L, C-Reaction protein 1.21mg/L, D-Dimer 1.13mg/L, the plasma protamine paracoagulation test was negative and infuse "suspended red blood cells 4iu + plasma 500ml" to prevent or correct anemia and supplement coagulation factors.

Through the analysis of the above treatment process, we ruled out the possibility of hemorrhage caused by the organic damage due to hysteroscope surgery. The patient’s blood coagulation function, the platelet count, coagulation function, D-dimer and inflammation indicators were all normal, and the 3P test was negative. The other three patients who underwent CS/UC-MSCs on the same day (September, 24,2019) used the same batch of UC-MSCs, and no adverse events occurred, indicating that this batch of stem cells is safe. We consider it may be abnormal uterine bleeding caused by ovulatory disorder.

In the afternoon of October 17, 2019, she was given 3.75 mg of norethindrone tablets orally every 6 hours as well as infection prevention treatment. Bleeding is significantly reduced on the same day, and stopped next day. The dose of norethindrone tablets was decreased one third every 3 days until 18 days later (November 5, 2019). Two weeks after the norethindrone was stopped, the patient still had no menstruation, so she underwent a B-ultrasound examination on November 19, 2019. The results showed that the endometrial thickness was 4mm, there were 4 small follicles on the right ovary and 3 small follicles on the left ovary. After that, the patient received hormone replacement therapy (estradiol valerate 2 mg two times a day for 18 days, then estradiol valerate was continued and progesterone therapy was then initiated progesterone soft capsule 200mg/day for 10 days, orally). 3 days after the therapy was stopped, the patient had menstruation with low amount on December 20,2019. The patient has a natural menstruation again on the February 11, 2020 and her menstrual volume was similar to that before the UC/UC-MSCs transplantation.


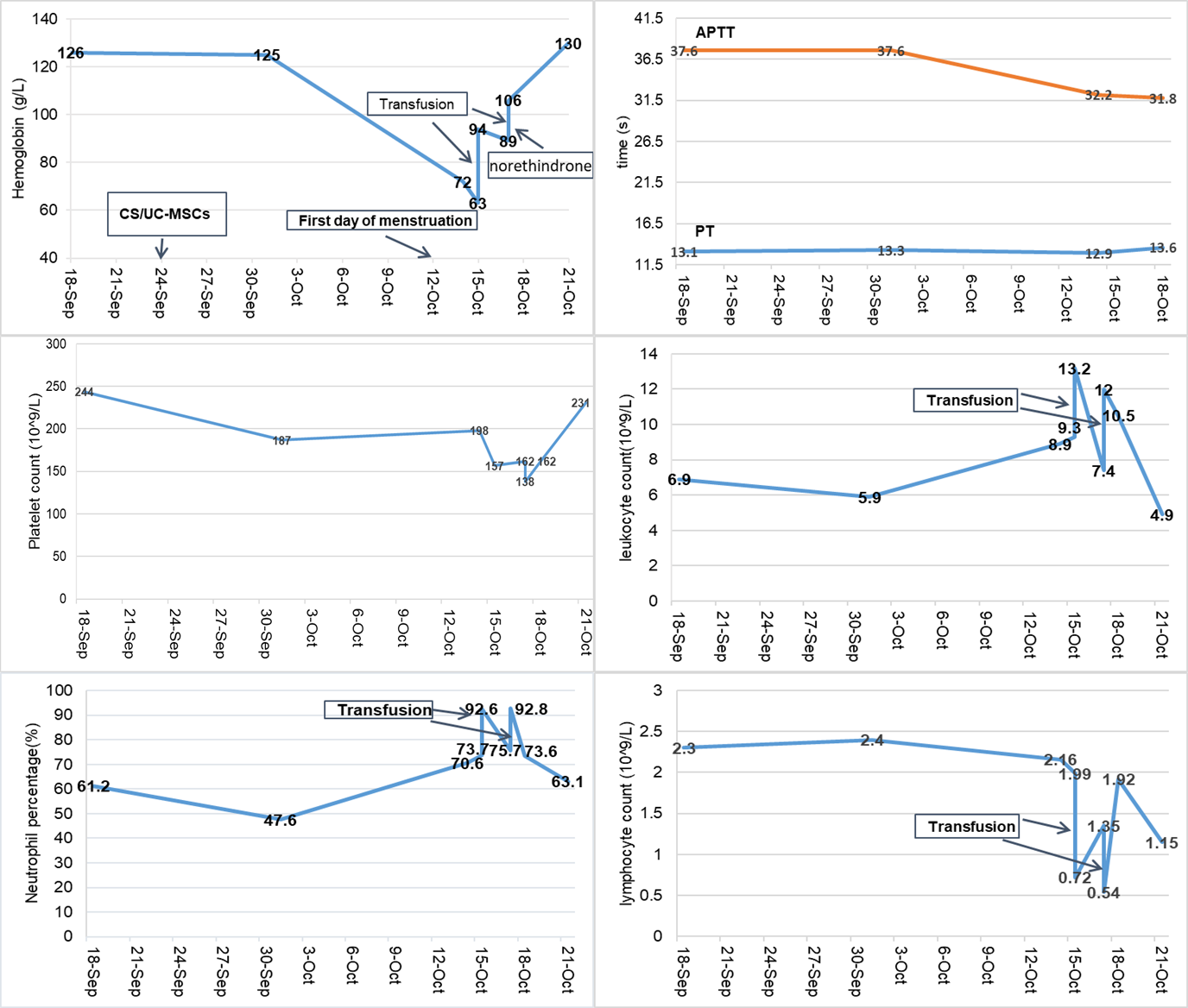


**A****fter multidisciplinary discussions among our team, we believed that “there was no direct evidence that this patient’s abnormal uterine bleeding was related to the stem cell therapy and the possibility of anovulatory abnormal uterine bleeding cannot be ruled out.” based on the following aspects:**

1. During the treatment, we performed hysteroscopy. Blood clots were seen in the uterine cavity, diffuse oozing blood was seen after removal of the clots, no obvious pulsatile bleeding and no residual collagen scaffold was seen. we ruled out the possibility of hemorrhage caused by the organic damage due to hysteroscope surgery.

2. During the treatment, except for the significant decrease in hemoglobin, the platelet count, coagulation function, D-dimer and inflammation indicators were all normal, and the plasma protamine paracoagulation test was negative. The possibility of bleeding caused by coagulation or infection was ruled out.

3. We rechecked the quality standard of the UC-MSCs given to this patient, no abnormal was found. The other three patients who underwent CS/UC-MSCs on the same day (September, 24,2019) used the same batch of UC-MSCs, and no adverse events occurred, indicating that this batch of stem cells is safe.

4. The patient's bleeding was significantly reduced after treatment with norethindrone and his symptoms improved. The menstruation was turned naturally afterwards with the same amount as before the treatment.
